# A Commensal *Helicobacter* sp. of the Rodent Intestinal Flora Activates TLR2 and NOD1 Responses in Epithelial Cells

**DOI:** 10.1371/journal.pone.0005396

**Published:** 2009-04-29

**Authors:** Nadia Chaouche-Drider, Maria Kaparakis, Abdulgader Karrar, Maria-Isabel Fernandez, Letitia A. M. Carneiro, Jérôme Viala, Ivo Gomperts Boneca, Anthony P. Moran, Dana J. Philpott, Richard L. Ferrero

**Affiliations:** 1 Institut Pasteur, Unité de Pathogénie Bactérienne des Muqueuses, Paris, France; 2 Department of Microbiology, Monash University, Clayton, Victoria, Australia; 3 Institut Pasteur, Unité de Pathogénie Microbienne Moléculaire, Paris, France; 4 INSERM U389, Paris, France; 5 Institut Pasteur, Groupe d'Immunité Innée et Signalisation, Paris, France; 6 Department of Immunology, University of Toronto, Toronto, Ontario, Canada; 7 Institut Pasteur, Groupe de Biologie et Génétique de la Paroi Bactérienne, Paris, France; 8 INSERM, Groupe Avenir, Paris, France; 9 Department of Microbiology, National University of Ireland, Galway, Ireland; Columbia University, United States of America

## Abstract

*Helicobacter* spp. represent a proportionately small but significant component of the normal intestinal microflora of animal hosts. Several of these intestinal *Helicobacter* spp. are known to induce colitis in mouse models, yet the mechanisms by which these bacteria induce intestinal inflammation are poorly understood. To address this question, we performed *in vitro* co-culture experiments with mouse and human epithelial cell lines stimulated with a selection of *Helicobacter* spp., including known pathogenic species as well as ones for which the pathogenic potential is less clear. Strikingly, a member of the normal microflora of rodents, *Helicobacter muridarum*, was found to be a particularly strong inducer of CXC chemokine (Cxcl1/KC, Cxcl2/MIP-2) responses in a murine intestinal epithelial cell line. Time-course studies revealed a biphasic pattern of chemokine responses in these cells, with *H. muridarum* lipopolysaccharide (LPS) mediating early (24–48 h) responses and live bacteria seeming to provoke later (48–72 h) responses. *H. muridarum* LPS per se was shown to induce CXC chemokine production in HEK293 cells stably expressing Toll-like receptor 2 (TLR2), but not in those expressing TLR4. In contrast, live *H. muridarum* bacteria were able to induce NF-κB reporter activity and CXC chemokine responses in TLR2–deficient HEK293 and in AGS epithelial cells. These responses were attenuated by transient transfection with a dominant negative construct to NOD1, and by stable expression of NOD1 siRNA, respectively. Thus, the data suggest that both TLR2 and NOD1 may be involved in innate immune sensing of *H. muridarum* by epithelial cells. This work identifies *H. muridarum* as a commensal bacterium with pathogenic potential and underscores the potential roles of ill-defined members of the normal flora in the initiation of inflammation in animal hosts. We suggest that *H. muridarum* may act as a confounding factor in colitis model studies in rodents.

## Introduction

Mouse models of “spontaneous colitis” have been reported to mimic the lesions observed in human inflammatory bowel disease (IBD) [1]. Though the underlying immunological defects conferring host susceptibility to colitis in these models vary, they all depend on the presence of an intestinal microflora for the initiation of disease [1]. The genus *Helicobacter* contains several intestinal species that have been linked with the development of colitis in mammalian hosts [2]. Two such species, *Helicobacter bilis* [3] and *Helicobacter hepaticus* [4], [5], have been shown to induce particularly severe forms of colitis in mice with defects in cytokine or T-cell functions, whereas *Helicobacter cinaedi* and *Helicobacter fennelliae* have been associated with colitis in humans [2] Many intestinal *Helicobacter* spp., however, do not have a clearly defined role in pathogenesis. One example is *Helicobacter muridarum*, which was initially described as a member of the normal flora of conventional rodents [6]. Subsequent studies, however, showed that *H. muridarum* could induce colitis and gastritis in mice, suggesting a potentially pathogenic role for the bacterium [7], [8], [9].

Intestinal epithelial cells have long been considered to act as a physical barrier that protects the integrity of epidermal or mucosal surfaces. A growing body of evidence now suggests an active role for these cells in host defense. Indeed, intestinal epithelial cells express several types of transmembrane pathogen recognition molecule (PRM) [10], [11]. Among the best characterized of these molecules are Toll-like receptor 2 (TLR2), which recognizes lipoprotein/lipoteichoic acid as well as atypical forms of lipopolysaccharide (LPS) [12], [13], and TLR4, which responds to the classical forms of Gram-negative LPS, such as that of *Escherichia coli*. Although PRMs are thought to play a crucial role in host immune responses to microbial pathogens, the mechanisms by which such molecules discriminate between pathogenic organisms and the host commensal microflora remain unclear. Unrestrained activation of intestinal innate immune molecules by micro-organisms plays a fundamental role in the pathophysiology of IBD [14].

In addition to the TLRs, a family of intracytoplasmic PRMs with homology to plant resistance proteins, known as the NOD-like receptors (NLRs) [15], has been described. Two members of this family, NOD1 (or CARD4) and NOD2 (or CARD15), were reported to respond to peptidoglycan, a component of bacterial cell walls [16], [17]. NOD1 displayed a high specificity for Gram-negative peptidoglycan [18]. Moreover, this molecule was implicated in epithelial cell responses to a variety of gastrointestinal bacteria, including the gastric pathogen *Helicobacter pylori* [15].

Host cell recognition of micro-organisms or their products via PRMs is known to initiate pro-inflammatory signaling events that converge on the transcription factor, nuclear factor-κB (NF-κB), culminating in up-regulated cytokine/chemokine gene expression in cells. Epithelial cell-derived CXC chemokines, such as CXCL8 (or interleukin-8), are critical mediators in the development of local inflammation in human IBD [19]. Mice do not possess a homolog of CXCL8, and produce instead Cxcl1 (KC) and Cxcl2 (MIP-2); these two chemokines are the major promoters of leukocyte recruitment in murine tissues [20], [21].

While the pathophysiology of *Helicobacter*-induced colitis in mouse models is well characterized, the inflammatory mediators and PRMs involved in the development of inflammation in these models is still poorly understood. We sought to address this question by studying NF-κB and/or CXC chemokine responses in epithelial cell lines stimulated with a selection of intestinal *Helicobacter* spp. This work has allowed us to identify, for the first time, the contribution of two PRMs, TLR2 and NOD1, in innate immune recognition of a commensal *Helicobacter* sp. of the normal rodent flora.

## Results

### Enterohepatic *Helicobacter* spp. induce CXC chemokine synthesis in murine intestinal epithelial cells

The effect of murine *Helicobacter* spp. on pro-inflammatory cytokine production in epithelial cells was evaluated in the mouse small intestinal cell line, m-IC_cl2_ [22]. For the purposes of this study, we chose two pathogenic species, *H. bilis* and *H. hepaticus* [3], [4], [5], and one species of ill-defined pathogenicity, *H. muridarum*.

**Figure 1:**
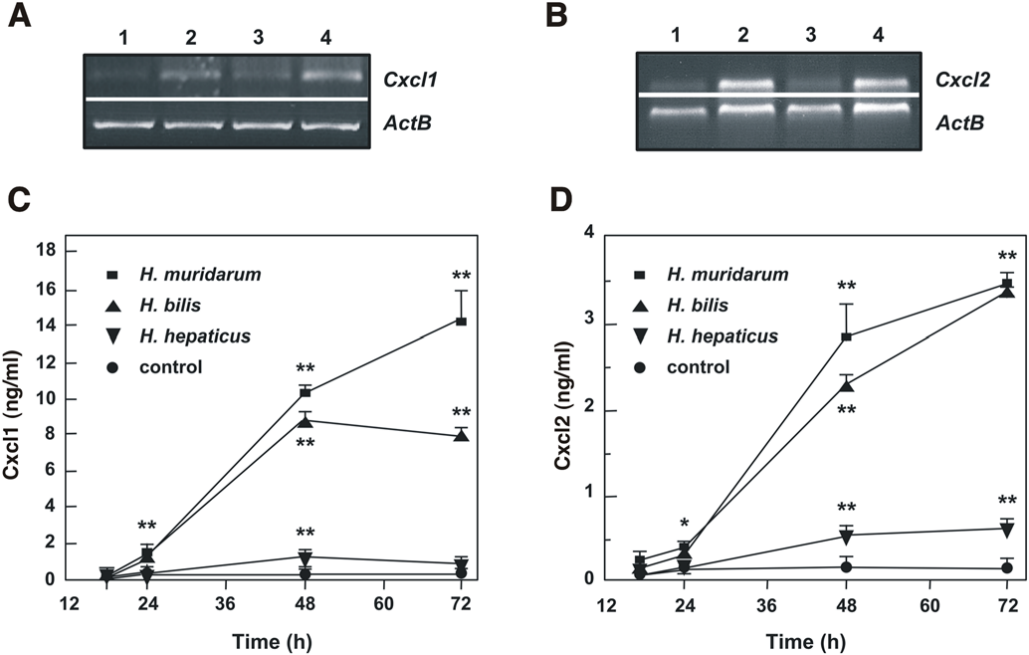
Intestinal *Helicobacter* spp. induce CXC chemokine responses in m-IC_cl2_ epithelial cells. (A) *Cxcl1* and (B) *Cxcl2* mRNA expression was detected by RT-PCR in (1) unstimulated cells and in cells stimulated for 18 h with (2) *H. bilis* ATCC51630, (3) *H. hepaticus* ATCC51448, or (4) *H. muridarum* ATCC49282. The quantities of total RNA were standardized by performing PCR with oligonucleotides specific for β-actin (*ACTB*). These data are representative of two independent experiments. The levels of secreted (C) Cxcl1 and (D) Cxcl2 were measured at the indicated times in the culture supernatants of control unstimulated m-IC_cl2_ cells, and cells stimulated with either *H. bilis* ATCC51630, *H. hepaticus* ATCC51448 or *H. muridarum* ATCC49282. Data correspond to the mean±SD (triplicate determinations) and are representative of three independent experiments. Statistical differences were observed between Cxcl1 or Cxcl2 levels following bacterial stimulation compared to control cells (*, *P*<0.03; **, *P*<0.001).

All three *Helicobacter* spp. induced increases in gene expression of the CXC chemokines, Cxcl1 and Cxcl2 (Figure 1A and 1B). *Cxcl1 and Cxcl2* mRNA levels were maximal at 18 h post-stimulation and were greatest in cells stimulated with either *H. bilis* ATCC51630 or *H. muridarum* ATCC49282. The levels of Cxcl1 and Cxcl2 steadily increased over time in *Helicobacter*-stimulated m-IC_cl2_ cells, with maximal levels present at between 48 and 72 h post-stimulation (Figure 1C and 1D). In agreement with the mRNA data, *H. muridarum* ATCC49282 and *H. bilis* ATCC51630 consistently induced 5- to 10-fold greater quantities of Cxcl1 and Cxcl2 production when compared to *H. hepaticus* ATCC51448. Interestingly, Sterzenbach and colleagues [23] also reported poor responses of m-IC_cl2_ cells to stimulation with live *H. hepaticus*. As previously described for LPS-stimulation [11], stimulation with *Helicobacter* bacteria did not appear to induce interleukin-6 nor tumor necrosis factor production in m-IC_cl2_ cells (data not shown).

### Cell contact-dependent chemokine responses in murine epithelial cells to live *H. muridarum* bacteria

**Figure 2:**
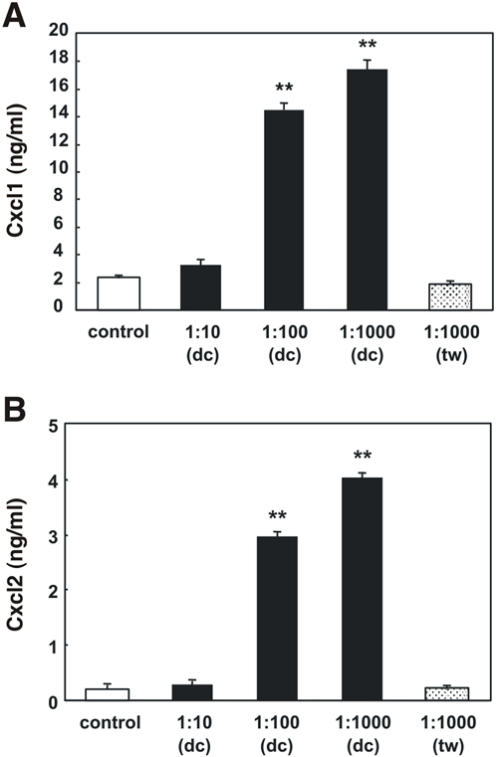
*H. muridarum* induces CXC chemokine production in m-IC_cl2_ cells by a contact-dependent mechanism. m-IC_cl2_ cells were either left unstimulated (control) or stimulated with different amounts (expressed as the MOI) of live *H. muridarum* ATCC49282 bacteria. The bacteria were incubated in direct contact (dc) or separated from the cells by a transwell (tw) filter membrane. The levels of secreted (A) Cxcl1 and (B) Cxcl2 were measured in culture supernatants at 48 h post-stimulation. Data correspond to the mean±SD (triplicate determinations). Statistical differences were observed between Cxcl1 or Cxcl2 levels following bacterial stimulation compared to control cells (**, *P*≤0.0002).

Given that *H. muridarum* induced consistently high levels of chemokine production in m-IC_cl2_ cells, and that its potential pathogenicity is poorly understood, we chose to further investigate the interactions of this *Helicobacter* sp. with epithelial cells. The first aim was to determine the role of cell-cell contact in *H. muridarum* induction of CXC chemokine synthesis in m-IC_cl2_ cells. The separation of *H. muridarum* ATCC49282 bacteria from m-IC_cl2_ cells by Transwell filter membranes was found to completely abrogate the Cxcl1 and Cxcl2 responses observed at 48 h post-incubation (Figure 2). Moreover, increasing the numbers of Transwell-separated *H. muridarum* bacteria to ten times those required in direct contact assays did not augment CXC chemokine production in the cells (Figure 2). These data demonstrated that direct bacterial-cell contact was required for epithelial cell responses at 48 h post-incubation with *H. muridarum* ATCC49282. Furthermore, the data suggest that it is unlikely that the responses observed at ≥48 h could be mediated by bacterial products released during bacterial lysis, as these products would not be retained by Transwell filters. In which case, the filters should not have had any effect on chemokine production by the cells.

### *H. muridarum* LPS induces pro-inflammatory responses in epithelial cells

**Figure 3:**
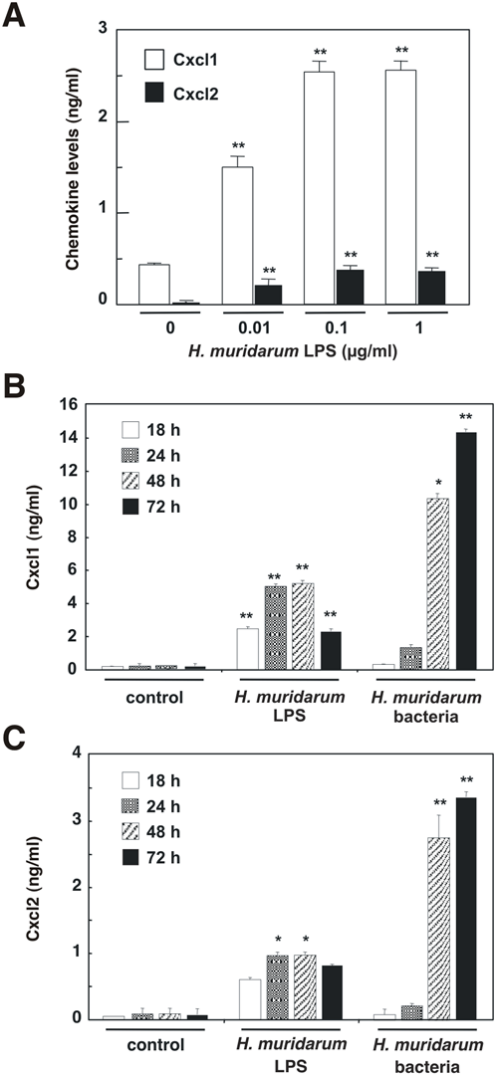
*H. muridarum* induces CXC chemokine production in m-IC_cl2_ cells by LPS-dependent and -independent mechanisms. m-IC_cl2_ cells were either left unstimulated (control) or stimulated with purified *H. muridarum* LPS or live *H. muridarum* ATCC49282 bacteria. The levels of secreted (A) Cxcl1 and Cxcl2 in the culture supernatants of cells stimulated for 48 h with different concentrations of *H. muridarum* LPS. Comparison of (B) Cxcl1 and (C) Cxcl2 levels in culture supernatants of cells stimulated for the indicated times with either 0.1 µg/ml *H. muridarum* ATCC49282 LPS or live *H. muridarum* ATCC49282 bacteria. Data correspond to the mean±SD (triplicate determinations) and are representative of three independent experiments. Statistical differences were observed between Cxcl1 or Cxcl2 levels following bacterial stimulation compared to control cells (*, *P* = 0.0002; **, *P*<0.0001).

It was previously reported that m-IC_cl2_ cells produce Cxcl2 in response to stimulation with *E. coli* LPS [11]. We therefore wished to determine whether *H. muridarum* LPS could induce similar responses in this cell line. Indeed, 0.1 µg/ml of highly purified *H. muridarum* ATCC49282 LPS was sufficient to induce a significant increase in Cxcl1 and Cxcl2 production in m-IC_cl2_ cells (*P*<0.0001; Figure 3A), with maximal responses detected at 24 h post-stimulation (Figure 3B and 3C). Interestingly, in contrast to the findings for purified LPS, live *H. muridarum* ATCC49282 bacteria induced significantly higher levels of Cxcl1 and Cxcl2 at the later time-points of 48 and 72 h (*P* = 0.0002 and *P*<0.0001; and *P*<0.0001 and *P*<0.0001, respectively; Figure 3B and 3C). Thus, the data suggested a biphasic pattern of responses in m-IC_cl2_ cells to *H. muridarum*.

**Figure 4:**
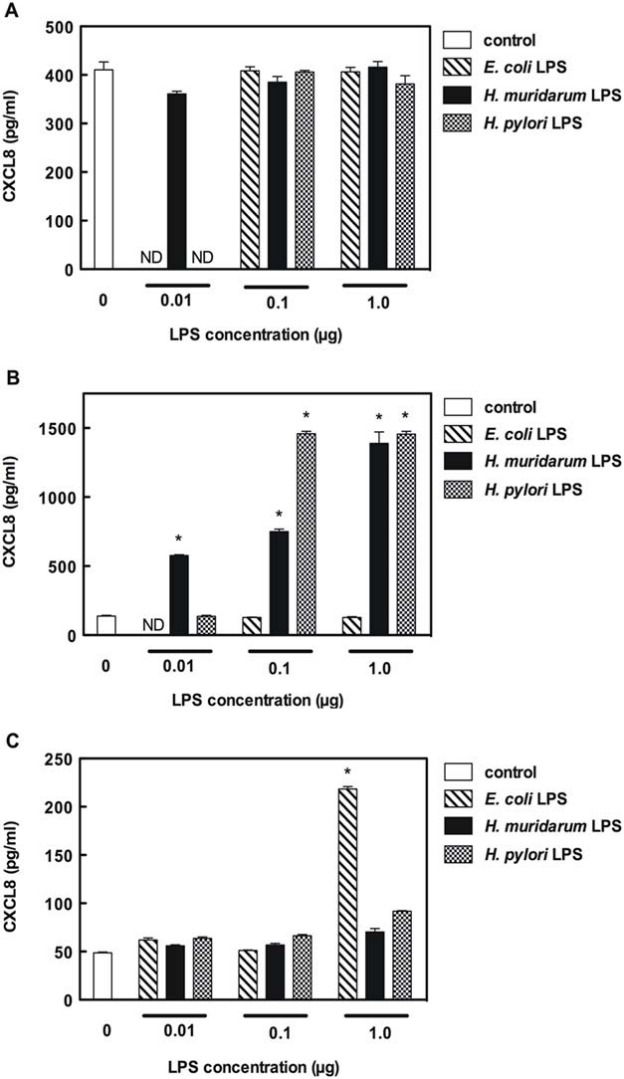
*H. muridarum* LPS is specifically recognized by TLR2. Normal HEK293 cells (A), as well as HEK293 cells stably expressing either TLR2 (B) or TLR4 (C), were either left untreated (control) or stimulated with highly purified LPS from *E. coli* 0111:B4, *H. muridarum* ATCC49282 or *H. pylori* NCTC11637. The levels of secreted CXCL8 were measured in culture supernatants at 24 h post-stimulation. Data correspond to the mean±SD (triplicate determinations) and are representative of two independent experiments. Statistical differences were observed between the CXCL8 responses of LPS-stimulated and control cells (*, *P*<0.0001).

Given that LPS appeared to be the key agonist involved in the early chemokine responses of m-IC_cl2_ cells to *H. muridarum* stimulation, we wished to determine the PRM involved in sensing of *H. muridarum* LPS. Using human embryonic kidney (HEK293) cell lines stably expressing either TLR2 or TLR4, we were able to show dose-dependent CXCL8 responses to *H. muridarum* ATCC49282 and *H. pylori* NCTC11637 LPS only in the TLR2-expressing cells (Figure 4). *E. coli* 0111:B4 LPS was used as a control in these experiments. As expected, HEK293 cells expressing exogenous TLR4 responded to this classical form of Gram-negative LPS, whereas no significant responses were detected in HEK293 cells, which are naturally deficient in either TLR2 or TLR4 [18], [24], nor in those expressing exogenous TLR2. As m-IC_cl2_ cells were shown to be responsive to the TLR2 agonist, Pam-3-Cys [11], it is likely that TLR2 was responsible for the early responses of these cells to *H. muridarum* LPS.

### Live *H. muridurum* bacteria induce NOD1-dependent responses in HEK293 and AGS cells

The studies in m-IC_cl2_ cell line suggested that an LPS-independent mechanism was likely to mediate the late chemokine responses to live *H. muridarum* bacteria (Figure 3B and 3C). To address this question, we used HEK293 cells which are naturally deficient in TLR2 and TLR4 expression [18]. As the HEK293 cell line is of human origin, we included in these experiments the human intestinal *Helicobacter* isolates, *H. cinaedi* and *H. fennelliae*.

**Figure 5:**
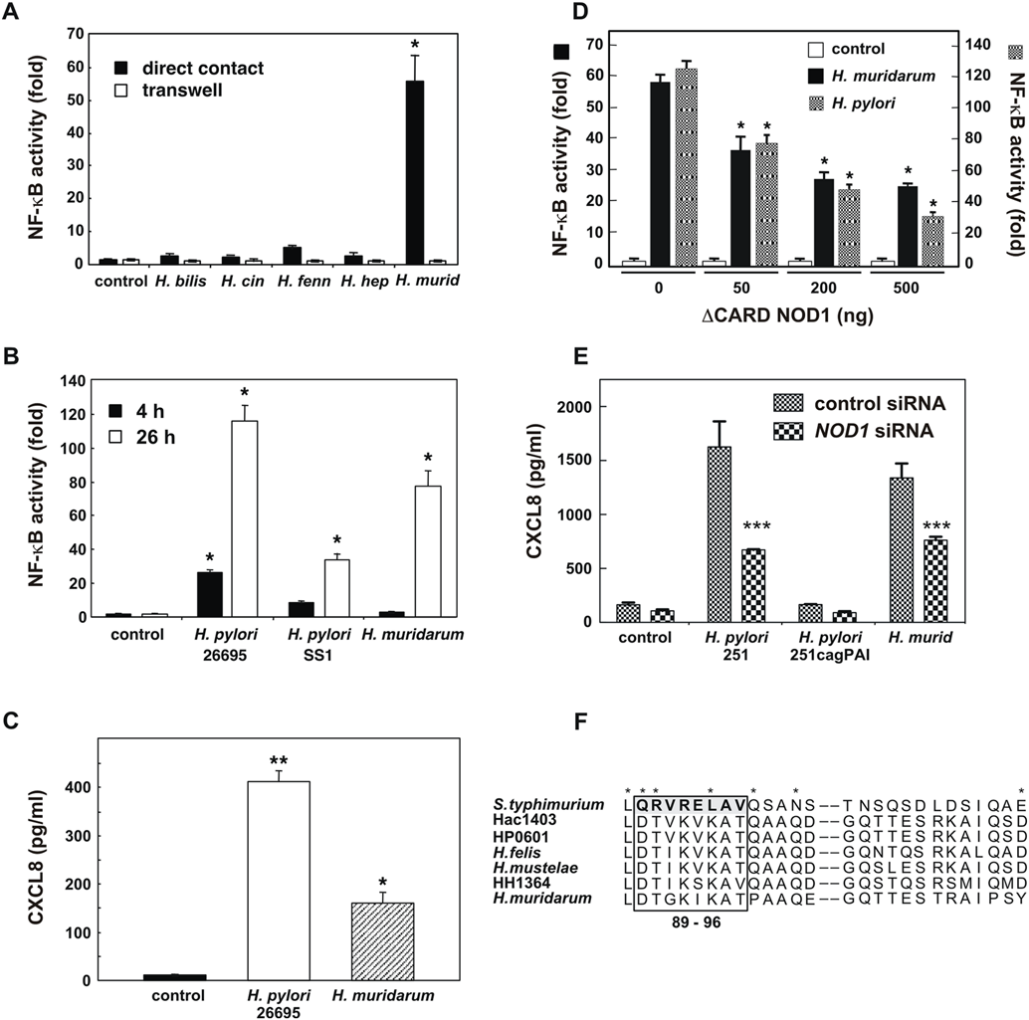
*H. muridarum* induces NOD1 responses in HEK293 and AGS cells. (A) NF-κB-dependent luciferase reporter activity was measured in HEK293 cells that had been incubated for 24 h in direct contact, or separated by a transwell filter membrane, from the following bacteria: *H. bilis* ATCC51630, *H. cinaedi* ATCC35683 (*H. cin*), *H. fennelliae* ATCC35684 (*H. fenn*), *H. hepaticus* ATCC51448 (*H. hep*), or *H. muridarum* ATCC49282 (*H. murid*). Control cells were transfected with the pCDNA3 vector. (B) NF-κB reporter activity in HE293 cells that had been incubated in direct contact for 4 h or 26 h with *H. pylori* (strains 26695 or SS1) or *H. muridarum* ATCC49282 bacteria. Control cells were transfected with the pCDNA3 vector. (C) CXCL8 secretion in culture supernatants of HEK293 cells at 24 h post-stimulation with *H. muridarum* ATCC49282 or *H. pylori* 26695. Control cells were left unstimulated. (D) HEK293 cells were co-transfected with the Igk-*luc* reporter plasmid and increasing amounts (in ng) of a NOD1 dominant-negative construct (ΔCARD NOD1), then either left untreated (control) or stimulated for 24 h with *H. muridarum* ATCC49282 or *H. pylori* 26695 bacteria. The total quantity of DNA was standardized throughout using pCDNA3 alone. (E) AGS cell lines stably expressing siRNA to an irrelevant gene (control siRNA) or to *NOD1* (*NOD1* siRNA) were either left untreated (control), or co-cultured for 1 h with *H. pylori* 251, 251 cagPAI (an isogenic mutant that lacks a functional T4SS) or *H. muridarum* ATCC49282. The medium was changed and the CXCL8 levels measured in supernatants at 24 h post-stimulation. NF-κB and CXCL8 data (triplicate determinations) are representative of 2–3 independent experiments. Statistical differences were observed for NF-κB reporter activity and CXCL8 responses in stimulated versus control cells (*, *P*<0.05; **, *P*<0.0001; ***, *P* = 0.002). (F) The amino acid sequence of a segment of *H. muridarum* ATCC49282 FlaA (Accession no. FM992115) was deduced by DNA sequencing and compared by CLUSTALW analysis to the corresponding regions of flagellin homologs from *S. typhimurium* [29], *Helicobacter acinonychis* (Hac1403; Accession no. CAK00134), *H. pylori* (HP0601), *Helicobacter felis* (*H. felis*; Accession no. Q9XB38), *Helicobacter mustelae* (*H. mustelae*; Accession no. P50612) and *H. hepaticus* (HH1364). The *H. pylori* and *H. hepaticus* FlaA sequences can be accessed at http://cmr.jcvi.org/cgi-bin/CMR/CmrHomePage.cgi. The amino acid residues, corresponding to residues 89–96 of *S. typhimurium* FliC, which have been identified as being critical for TLR5 activation [29], are highlighted by a box. This region shares three amino acid residues with another series (shown by asterisks) that had previously been shown to be important for TLR5 signaling [31].

*H. muridarum* ATCC49282 induced a significant increase in NF-κB reporter activity in HEK293 cells, when compared to unstimulated cells (Figure 5A; *P*<0.05). Similar to the findings for the m-IC_cl2_ cell line, HEK293 responses to live *H. muridarum* were cell contact-dependent (Figure 5A). These responses could not, however, be attributed to the invasive properties of *H. muridarum* as this bacterium was found to be no more invasive than either *H. bilis* or *H. hepaticus* (Figure S1). Strikingly, the various other intestinal *Helicobacter* spp. tested here, including the human isolates *H. cinaedi* ATCC35683 and *H. fennelliae* ATCC35684, were very weak inducers of NF-κB reporter activity in HEK293 cells (Figure 5A).

HEK293 cells are able to respond to bacterial pathogens via the cytosolic PRM, NOD1 [15]. As *H. pylori* strains carrying a functional type IV secretion apparatus (T4SS) were shown to trigger NOD1 signaling in these cells [25], [26], we co-cultured HEK293 cells with either *H. muridarum* ATCC49282, or *H. pylori* bacteria possessing or not a functional T4SS (strains 26695 and SS1, respectively) [26]. *H. muridarum* ATCC49282 was found to induce similar levels of NF-κB-dependent reporter activity, albeit at a slower rate, than *H. pylori* bacteria with a functional T4SS (strain 26695; Figure 5B). Consistent with the NF-κB reporter studies, *H. muridarum* also up-regulated CXCL8 synthesis in HEK293 cells (Figure 5C). Transfection of these cells with increasing concentrations of a dominant-negative NOD1 construct, in which the caspase activation recruitment domain (CARD) was deleted [27], significantly abrogated *H. muridarum* effects on NF-κB reporter activity (Figure 5D; *P*<0.05). Furthermore, we demonstrated that AGS cells stably expressing siRNA to NOD1 produced significantly less CXCL8 in response to stimulation with *H. muridarum* (or *H. pylori*), when compared to cells expressing siRNA to an irrelevant control gene (Figure 5E; *P* = 0.002). The abrogation of NOD1 signaling by ΔCARD NOD1 or NOD1 siRNA resulted in comparable decreases in cell responses to either *H. muridarum* ATCC49282 or T4SS-positive *H. pylori*. Thus, the data suggested that NOD1 was important for epithelial cell responses to *H. muridarum*. As m-IC_cl2_ cells express NOD1 mRNA ([23]; Figure S2), we propose that NOD1 contributed to the LPS-independent responses to live *H. muridarum* bacteria in this cell line (Figure 3B and 3C). To exclude a role for NOD1 in the observed responses of m-IC_cl2_ cells to purified *H. muridarum* LPS (Figure 3), we delivered the purified material into the cytosol of HEK293 cells, together with NOD1-expressing construct, using a previously described liposome-based technique [18], [26]. Transfection of cells with *H. muridarum* ATCC49282 LPS was shown to have no effect on NF-κB activity, whereas *H. pylori* 26695 peptidoglycan transfected in this way induced a significant response (Figure S3). This suggests that the *H. muridarum* LPS preparation was devoid of peptidoglycan muropeptides, which might otherwise induce NOD1 signaling in m-IC_cl2_ cells. It also suggests that the observed activity of *H. muridarum* LPS activity on TLR2 signaling (Figure 4) could not be ascribed to lipoprotein contamination of this LPS preparation.

Although the data strongly suggested that NOD1 is a key PRM involved in epithelial cell responses to *H. muridarum*, HEK293 and AGS cells both also express the PRM for bacterial flagellin, TLR5 [26], [28]. All *Helicobacter* spp. are flagellated and it was therefore possible that this PRM may be involved in the cell responses to *H. muridarum.* To examine this suggestion, we sequenced a region of the gene encoding the major flagellin subunit (FlaA) of *H. muridarum* ATCC49282 (Figure 5F). We demonstrated that *H. muridarum* FlaA does not possess any of the eight highly conserved amino acid residues, corresponding to residues 89–96 of *Salmonella typhimurium* FliC, which have been found to be required for activation of TLR5 by bacterial flagellins [29], [30], [31]. Instead, the corresponding region of *H. muridarum* FlaA displayed a high degree of conservation with the FlaA homologs of gastric and intestinal *Helicobacter* spp. which are known to evade TLR5 recognition [23], [29], [30]. These findings, together with the lack of a systematic responsiveness of epithelial cells to other flagellated *Helicobacter* spp. (Figure 5A; [23], [29]), also allow us to formally exclude TLR5 as playing a significant role in *H. muridarum* recognition.

## Discussion

Enterohepatic *Helicobacter* spp. have been reported to induce chronic intestinal inflammation in animal hosts [2], [3], [7]. The role of these bacterial species in the development of intestinal inflammation is, however, poorly understood. By studying epithelial cell responses to various enterohepatic *Helicobacter* spp. of murine and human origin, we showed that *H. muridarum*, an isolate from the normal intestinal flora of conventional rodents [6], [9], was capable of inducing CXC chemokine production and NF-κB activation in epithelial cells *in vitro*. Collectively, this bacterium was found to be a more potent inducer of pro-inflammatory responses in mouse and human cell lines than either of the two mouse pathogens, *H. bilis* or *H. hepaticus*. Epithelial cell responses to *H. muridarum* were mediated by both LPS-dependent and -independent mechanisms. We propose that the intracellular PRM, NOD1, participates in the LPS-independent responses to *H. muridarum*. While there have been several reports concerning NOD1 signaling to gastrointestinal pathogens [15], this is the first describing the involvement of this molecule in epithelial cell responses to a member of the normal microbial flora.

*H. muridarum* was originally cultivated from the ileal and cecal tissues of conventional rodents [6]. *H. muridarum* was also reported to colonize the gastric mucosa of aged conventional mice, and to induce gastritis in these animals [8], [9]. Moreover, mice that were monoassociated with *H. muridarum* displayed an accelerated development of IBD-like lesions in a CD45RB^high^ CD4^+^ T cell transfer model of experimental colitis [7]. Hence, it was suggested that the bacterium was a “provocateur” of IBD [7]. Consistent with this suggestion, *H. muridarum* was shown here to induce robust pro-inflammatory responses in epithelial cells of human and mouse origins. Co-culture of m-IC_cl2_ mouse intestinal cells with *H. muridarum* resulted in increased transcription and synthesis of two CXC chemokines, Cxcl1 and Cxcl2. These chemokines are key promoters of polymorphonuclear leukocyte recruitment in mice [20], [21] and may thus represent important mediators of the inflammatory responses observed in *Helicobacter* colitis models.

The m-IC_cl2_ cell line synthesizes functional TLR2 and TLR4 molecules [11], [23]. Stimulation of these cells with either TLR2 or TLR4 ligands (Pam-3-Cys and LPS, respectively) resulted in increased NF-κB-dependent pro-inflammatory cytokine production [11], [32]. In the current study, we demonstrated a dose-dependent effect of *H. muridarum* LPS on Cxcl1 and Cxcl2 synthesis in m-IC_cl2_ cells (Figure 3A). These responses appear to depend on TLR2 recognition of this LPS (Figure 4). Interestingly, as found here (Figure 4B) and elsewhere [12], [13], *H. pylori* LPS also seems to signal via TLR2, however the situation is not clear as other researchers have claimed TLR4 to be the cognate PRM for this LPS molecule [33]. These differences may be attributed to the experimental conditions (*e. g*. LPS concentrations) used in the various studies.

Although m-IC_cl2_ cells were highly responsive to *H. muridarum* LPS, the kinetics and magnitude of these responses were different to those induced by live bacteria (Figure 3B and 3C). This finding suggested the existence of an LPS-independent mechanism in epithelial cell signaling to *H. muridarum.* Further experiments were undertaken in the HEK293 and AGS cell lines, neither of which express functional TLR2 nor TLR4 [18]. Of the five murine and human *Helicobacter* spp. tested, only *H. muridarum* had a dramatic effect on NF-κB reporter activity in HEK293 cells (Figure 5A). Preliminary studies with three other *H. muridarum* strains suggest that it may be a strain-independent phenomenon (data not shown). Although *H. bilis*, and to a lesser extent, *H. hepaticus*, induced CXC chemokine production in m-IC_cl2_ cells, these species were poor agonists of NF-κB responses in HEK293 cells. Taken together, the findings highlight the specific nature of HEK293 cell responses to *H. muridarum*. Moreover, responses to *H. muridarum* were demonstrated to be NOD1-dependent and TLR5-independent (Figure 5). The latter finding is consistent with the conclusions of several studies reporting that TLR5 is not important for epithelial cell recognition of *Helicobacter* spp. [23], [29], [30].

The present work raises two important questions relating to the bacterial pathogenesis of enterohepatic *Helicobacter* spp. The first of these concerns the mechanism by which *H. muridarum* may trigger NOD1 signaling in epithelial cells. NOD1 responds specifically to Gram-negative peptidoglycan presented within the cytoplasm of epithelial cells by pathogenic bacteria. Until now, this has been reported to occur via either cell invasion or by the actions of a bacterial T4SS, encoded by the *H. pylori cag* pathogenicity island (PAI) [15]. *H. muridarum*, however, does not appear to be any more invasive than other *Helicobacter* spp (Figure S1) [26], and does not harbor the genes needed to encode a functional T4SS (RLF, unpublished data). Thus, *H. muridarum* is likely to utilize a different mechanism of peptidoglycan delivery than those described previously. This suggestion would be consistent with the relatively longer incubation periods (>24 h) required for *H. muridarum*-induced NF-κB reporter activity in HEK293 cells, when compared to those used for the T4SS-positive *H. pylori* 26695 strain (≤4 h; Figure 5B). In AGS cells, however, a 1 h contact time with cells was sufficient for the induction of responses by *H. muridarum*, whereas this was insufficient for *H. pylori* 251*cagPAI*, a T4SS-deficient mutant (Figure 5E). Nevertheless, when compared to T4SS-positive *H. pylori*, 10-fold more *H. muridarum* bacteria (MOI 1∶100) were required to induce comparable responses in these cells, suggesting that *H. muridarum* is a less efficient initiator of NOD1 signaling. These findings point towards the existence in *H. muridarum* of an alternative mechanism for activation of the NOD1 pathway.

A second question arising from the work relates to the low pro-inflammatory activity of *H. hepaticus* on epithelial cell lines. Although this bacterium has been shown to induce colitis in certain strains of mice [4], [5], one study reported that germ-free IL-10^−/−^ mice monoassociated with *H. hepaticus* (the same strain as the one used here) did not develop colitis [34]. It is thus possible that the presence of a complex intestinal flora may promote the virulence potential of *H. hepaticus*. It has also been suggested that the level of pathogenicity exhibited by different *H. hepaticus* isolates may be strongly influenced by strain-specific differences in gene content, including the presence or otherwise of a complete PAI [35], [36]. Conversely, there have been reports that under certain circumstances *H. hepaticus* is able to dampen inflammation [37]. Consistent with this suggestion, Sterzenbach *et al*. showed that m-IC_cl2_ cells that had been pre-incubated with *H. hepaticus* cell lysates, responded poorly to TLR4 and TLR5 agonists [23]. Nevertheless, both that work as well as our own were performed in relatively simple epithelial cell models that mimic only a small aspect of the complex interactions occurring between host cells and intestinal pathogens or commensals. Thus, even if *H. hepaticus* may be able to down-regulate pro-inflammatory responses in epithelial cells, it is plausible that its interactions with other mucosal cell populations and/or other components of the host microflora may contribute to the inflammation normally associated with infection by this bacterium.

Mazmanian and colleagues [38] hypothesized that intestinal inflammation may be caused by imbalances between commensal organisms that have a pathogenic potential (so-called “pathobionts”) and those with beneficial potential (“symbionts”). From the evidence presented here, it appears that *H. muridarum* has a pathogenic potential and should therefore be considered a pathobiont. This observation has practical consequences for those who work with rodent models of inflammation and immunity. Indeed, *H. muridarum* may be “missed” by the assays commonly employed to monitor the specific pathogen-free (SPF) status of animals. Furthermore, although some laboratory animal suppliers use *Helicobacter* genus-specific PCR assays to verify the SPF status of their animals, this is not always the case. *H. muridarum* (or indeed other poorly studied *Helicobacter* spp.) may therefore have a confounding effect in mouse colitis models, particularly as *H. muridarum* was shown to engage TLR2 and NOD1, both of which are expressed in intestinal epithelial cells [11], [23], [39], [40]. Given that defects in TLR and/or NLR signaling are thought to affect the ability of host cells to respond normally to the intestinal microflora, it is plausible that these together with imbalances within the flora may contribute to the development of inflammatory conditions in the gut. Further investigations should thus be undertaken to elucidate the role of PRMs in epithelial cell sensing of members of the normal microflora.

## Materials and Methods

### Bacteria

*H. bilis* (CIP204753T, ATCC51630), *H. cinaedi* (CIP103752T, ATCC35683); *H. hepaticus* (ATCC51448), *H. fennelliae* (ATCC35684); and *H. muridarum* (St1, ATCC49282) were kindly provided by the Culture Collection of the Institut Pasteur (Paris, France), Prof. J. G. Fox (Massachusetts Institute of Technology, Boston, MA) and Dr J. O'Rourke (The University of NSW, Sydney, Australia), respectively. *H. pylori* 26695, SS1, and 251 strains were as described previously [26]. The *H. pylori* 251 *cag*PAI deletion mutant was constructed by natural transformation using the kanamycin resistance cassette developed by Odenbreit *et al.* [41].

Bacteria were routinely subcultured on Blood Agar Base No. 2 (Oxoid, Hampshire, UK), supplemented with 8–10% horse blood and an antibiotic cocktail [26]. Broth cultures were prepared in 10 ml Brain Heart Infusion (Oxoid) containing 10% heat-inactivated fetal calf serum (FCS; Invitrogen, Cergy-Pontoise, France and Auckland, New Zealand) [26]. Bacterial suspensions were washed three times in phosphate-buffered saline (pH 7.4), then resuspended in cell culture medium. Viable counts of the bacterial suspensions were determined by serial plating [26]. Highly purified *H. muridarum* ATCC49282 and *H. pylori* NCTC11637 LPS were prepared by hot phenol-water extraction and subsequent enzymatic treatments with DNase, RNase and proteinase K (Sigma Chemical Co., St Louis, MO) and by ultracentrifugation [42]. *E. coli* 0111:B4 Ultrapure LPS was obtained from InvivoGen (Toulouse, France).

### Cell culture conditions

The murine m-IC_cl2_ epithelial cell line represents a clone of immortalized cells that were derived from the bases of small intestinal villi of transgenic mice [22]. These cells were routinely grown in a supplemented DMEM-F12 medium (Invitrogen) [22]. Diluted cell suspensions were seeded onto collagen-coated (2 mg/ml rat tail collagen type 1, Sigma) culture plates, and incubated at 37°C in 5% CO_2_. For co-culture experiments, m-IC_cl2_ cells were grown for 1 day then serum-starved overnight. The cells were washed three times in DMEM-F12 medium, and re-incubated in antibiotic-free, serum-free medium, prior to addition of the bacteria or purified *H. muridarum* LPS. Culture supernatants were collected at the indicated times and frozen at −80°C until assayed.

HEK293 cells, as well as the TLR2- and TLR4-expressing HEK293 cell lines [43], were grown routinely in DMEM (Invitrogen) containing 10% FCS, and incubated at 37°C in 5% CO_2_ [25]. Bacteria were separated from epithelial cells using 0.2 µm Transwell filters (Corning Incorporated, Corning, NY). *Helicobacter* and *E. coli* LPS (0.01–1.0 µg) were added directly to HEK293 cells and the culture supernatants collected 8 h later.

AGS cells were routinely cultured in serum-supplemented RPMI 1640 cell culture media [25]. The AGS control siRNA and *NOD1* siRNA cells were generated by integration of an expression vector containing a small interference RNA (siRNA) directed to either the gene encoding enhanced green fluorescent protein (EGFP) or the CARD of the *NOD1* gene, respectively. A detailed description and characterization of these cell lines is given elsewhere (Grubman *et al*.; manuscript submitted). For co-culture assays, bacteria were added for 1 h to serum-starved cells (MOI 1∶10 for *H. pylori* and 1∶100 for *H. muridarum*), washed off, and the media replaced. Culture supernatants were collected 17–23 h later.

### Cell transfection assays

HEK293 cells were plated in 24-well plates at a density of 1×10^5^ cells and transfected the following day with Igκ-luciferase reporter DNA, using FuGene6 reagent medium (Roche Diagnostics, Meylan, France) [44]. For dominant-negative studies, cells were co-transfected with ΔCARD NOD1 DNA [27]. The transfected cells were incubated overnight and co-cultured with live bacteria (MOI = 1∶10 to 1∶100) for 2–24 h, prior to lysis of the cells [26]. Lysed cells were assayed for luciferase activities using a 96-well luminometer.

### Reverse Transcriptase–PCR (RT–PCR)

Total RNA was isolated from epithelial cells using TRIzol reagent (Invitrogen). RNA (1 mg) from each sample was reverse-transcribed using 25 U of superscript II reverse transcriptase (Invitrogen). PCR was conducted using 2 ml of cDNA and 0.25 U Taq DNA polymerase (Amersham Biosciences, Orsay, France). Fifteen pmol each of 5′ and 3′ primers for cytokine genes were used with 3 pmol each of β-actin primers in a multiplex reaction. One PCR cycle consisted of the following: 94°C for 1 min, 64°C for 1 min 15 s, and 72°C for 1 min 15 s. The total cycle numbers were 35. A final elongation step of 7 min at 72°C was then used. Primer sequences were as follows: murine *Cxcl1*, [45], 5′-TTGAAGGTGATGCCGCCAG-3′ and 5′-CCCAGACTCTCATCTCTCC-3′; murine *Cxcl2*, [46], 5′-CATCGAATTCGGCAGACTCCAGCCACACTTCAGCCT-3′ and 5′-GATCGGATCCGGCAGTTAGCCTTGCCTTTGTTCAGT-3′; murine β-actin (*ActB*), 5′-CCAGAGCAAGAGAGGTATCC-3′ and 5′-CTGTGGTGGTGAAGCTGTAG-3′. PCR products (with sizes of 205, 358 and 436 bp, respectively) were separated on 1.5% agarose gels with 0.4 mg/ml ethidium bromide. Stained bands were visualized under UV light, and photographed with an Image Master VDS machine (Amersham).

### PCR amplification of *H. muridarum flaA*

PCR was performed on genomic DNA (1–10 ng) that had been purified from *H. muridarum* using the MasterPure™ DNA Purification Kit (EPICENTRE Biotechnologies, Madison, WI). DNA samples from *H. pylori* 26695 and *H. hepaticus* ATCC51448 were used as positive controls. The PCR samples contained 20 µM of each primer, 200 µM of total dNTPs (Promega, Alexandria, NSW, Australia), 0.5 U Taq Polymerase Taq and buffer (both from Roche Applied Science, Castle Hill, NSW, Australia). One PCR cycle consisted of the following: 94°C for 1 min, 50°C for 1 min, and 72°C for 1 min 15 s. The total cycle numbers were 30. A final elongation step of 7 min at 72°C was used. Primer sequences were as follows: oligo A, 5′-ATGGCTTTTCAGGTCAATAC-3′; oligo C, 5′-CCTACTTGGAATTCTTTG-3′. PCR products (449 bp) were separated on 1.5% agarose gels, as above, and the DNA purified using the Perfectprep Gel Cleanup kit (Eppendorf North Ryde, NSW, Australia). DNA sequencing of the *H. muridarum flaA* amplicon was performed by Micromon Services (Monash University, Clayton, VIC, Australia) using BigDye Terminator Cycle Sequencing (Applied Biosystems, Scoresby, VIC, Australia). The deduced *H. muridarum* FlaA sequence was aligned with those of *S. typhimurium FliC* and the FlaA homologs of other *Helicobacter* spp. using CLUSTALW software (http://www.ebi.ac.uk/Tools/clustalw2/index.html).

### Chemokine assays

CXC chemokine (Cxcl1, Cxcl2 or CXCL8) levels were determined from culture supernatants using cytokine enzyme-linked immunosorbent assay (ELISA) kits from either R&D Systems (Minneapolis, MN) or BD Pharmingen (CA).

### Statistical analysis

Data were analyzed using the Student's t-test and Mann-Whitney test, as appropriate. Differences in data values were considered significant for *P*≤0.05.

## Supporting Information

Figure S1 Invasion efficiency of enterohepatic *Helicobacter* spp in HEK293 cells. The invasion efficiency of each bacterium was determined by the gentamycin protection assay [26]. The values are expressed as the proportions (in percent) of internalized bacteria to the total numbers in the inocula added to cells. (n = 2 independent experiments for *H. muridarum* and *H. bilis*; n = 1 for *H. hepaticus*.)
(1.96 MB DOC)

Figure S2 m-IC_cl2_ epithelial cells express Nod1 mRNA. RT-PCR detection of Nod1 (CARD4) mRNA expression in unstimulated m-IC_cl2_ epithelial cells (1) and in cells co-cultured for 18 h with either (2) *H. muridarum* or (3) mouse TNF. RNA samples were standardized by performing PCR with oligonucleotides specific for β-actin (ACTB) (see Materials and Methods). Amplicons (303 bp) from murine Nod1 (CARD4) were amplified using the following oligonucleotides: 5′-AGGAGGCCAACAGACGCC-3′ and 5′-CTGACCTAGAGGGTATCG-3′.
(2.59 MB TIF)

Figure S3 H. muridarum LPS does not induce NOD1 signaling in HEK293 cells. NF-κB responses of unstimulated HEK293 cells (control), unstimulated cells that had been transfected with a NOD1-expressing construct (control+NOD1), or in cells co-transfected with the NOD1-expressing construct as well as either *H. pylori* peptidoglycan (Hp PG+NOD1) or *H. muridarum* LPS (Hm LPS+NOD1). Data correspond to the mean±SEM (triplicate determinations) and are representative of two independent experiments. Statistical differences were observed between control cells and those stimulated with Hp PG+NOD1 (*, P<0.05).
(7.83 MB TIF)
