# Supplementary figures and images for: A Commensal Helicobacter sp. of the Rodent Intestinal Flora Activates TLR2 and NOD1 Responses in Epithelial Cells
Source: PLoS One. 2009 Apr 29;4(4):e5396. doi: 10.1371/journal.pone.0005396 (PMC2671595; doi:10.1371/journal.pone.0005396)

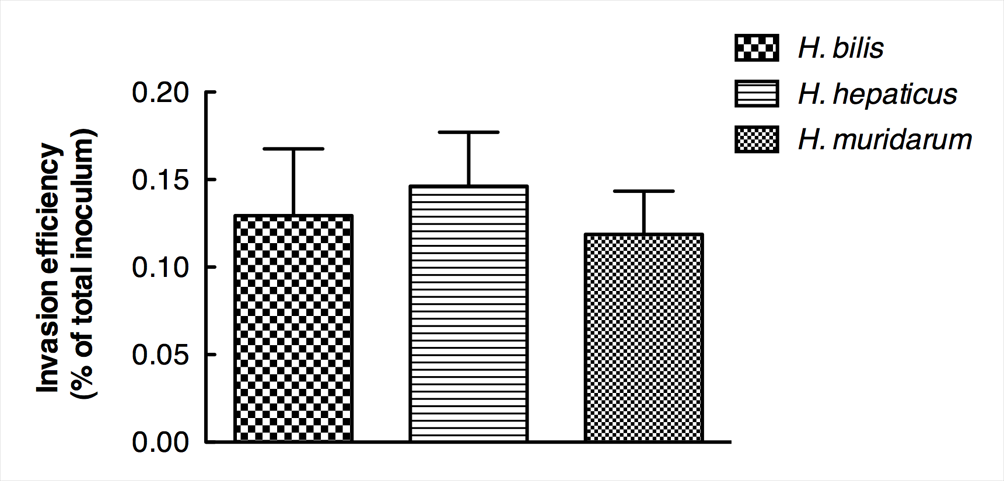

Supplement: Figure S1 — Invasion efficiency of enterohepatic Helicobacter spp in HEK293 cells. The invasion efficiency of each bacterium was determined by the gentamycin protection assay [26]. The values are expressed as the proportions (in percent) of internalized bacteria to the total numbers in the inocula added to cells. (n = 2 independent experiments for H. muridarum and H. bilis; n = 1 for H. hepaticus.) (1.96 MB DOC) [file pone.0005396.s001.doc]

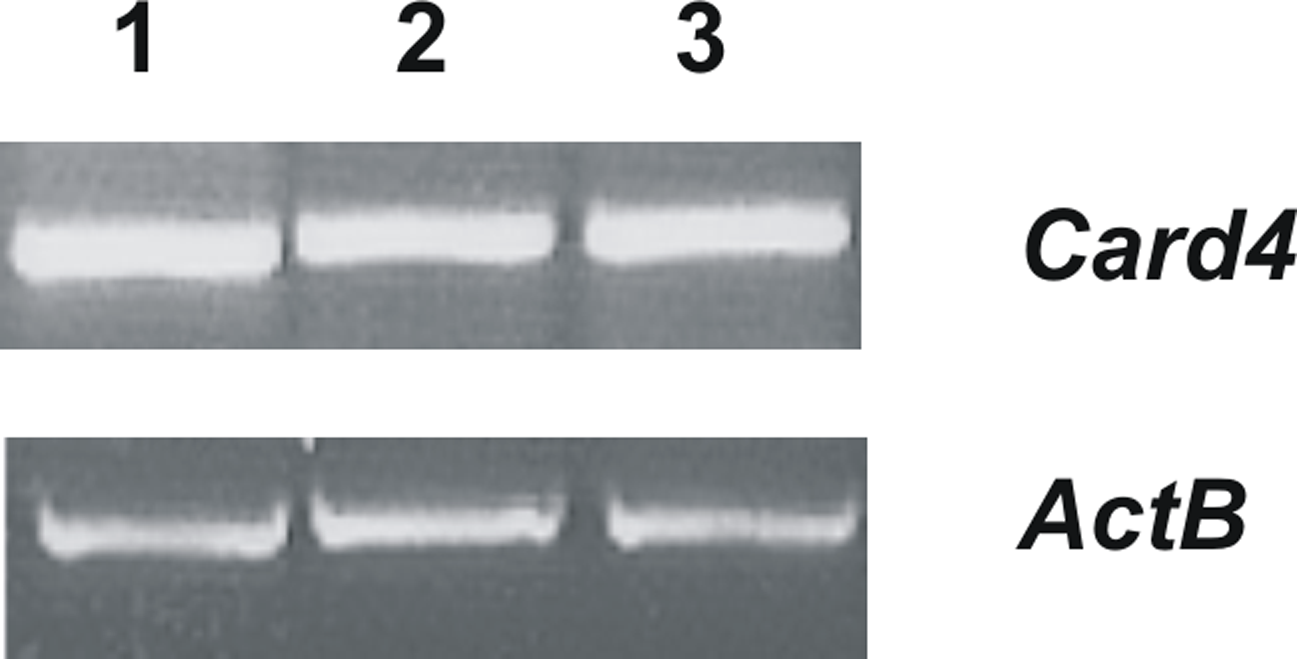

Supplement: Figure S2 — m-ICcl2 epithelial cells express Nod1 mRNA. RT-PCR detection of Nod1 (CARD4) mRNA expression in unstimulated m-ICcl2 epithelial cells (1) and in cells co-cultured for 18 h with either (2) H. muridarum or (3) mouse TNF. RNA samples were standardized by performing PCR with oligonucleotides specific for β-actin (ACTB) (see Materials and Methods). Amplicons (303 bp) from murine Nod1 (CARD4) were amplified using the following oligonucleotides: 5′-AGGAGGCCAACAGACGCC-3′ and 5′-CTGACCTAGAGGGTATCG-3′. (2.59 MB TIF) [file pone.0005396.s002.tif]

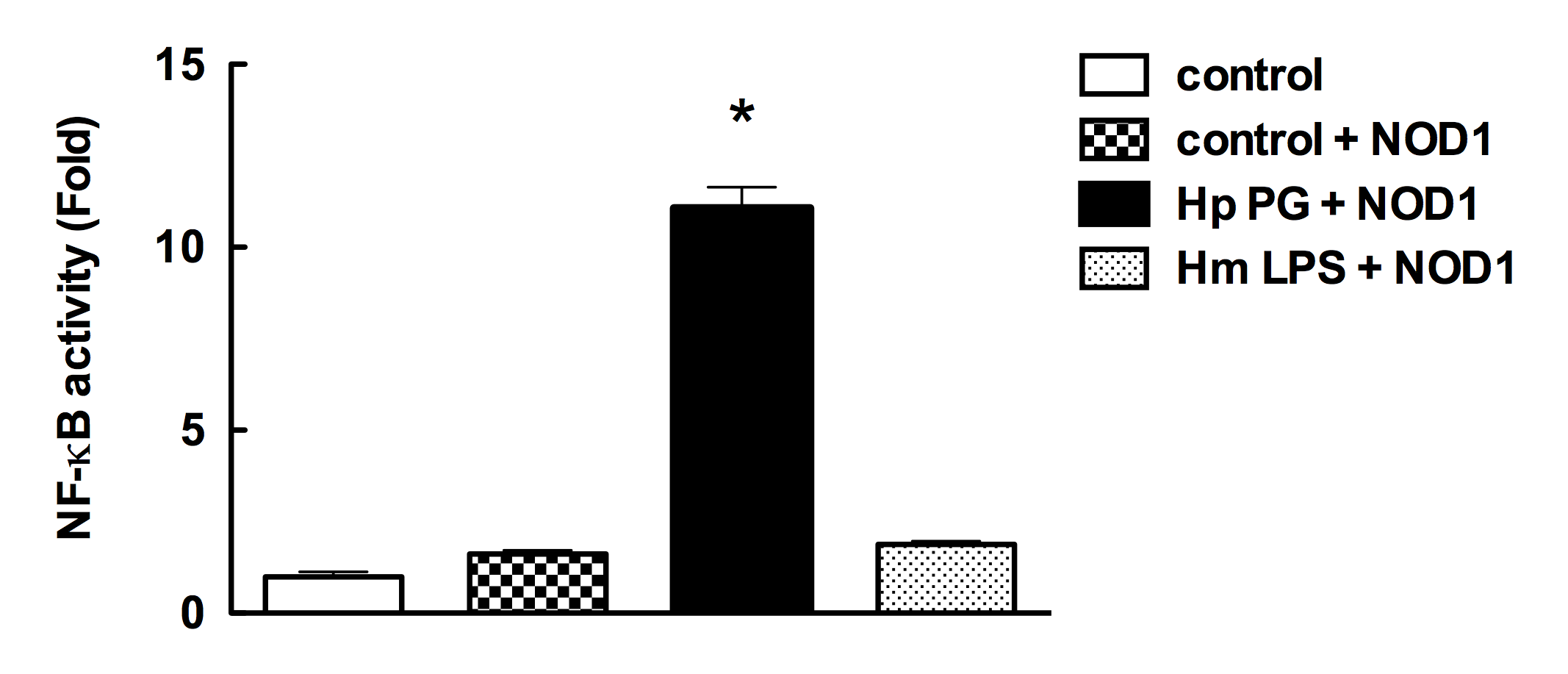

Supplement: Figure S3 — H. muridarum LPS does not induce NOD1 signaling in HEK293 cells. NF-κB responses of unstimulated HEK293 cells (control), unstimulated cells that had been transfected with a NOD1-expressing construct (control+NOD1), or in cells co-transfected with the NOD1-expressing construct as well as either H. pylori peptidoglycan (Hp PG+NOD1) or H. muridarum LPS (Hm LPS+NOD1). Data correspond to the mean±SEM (triplicate determinations) and are representative of two independent experiments. Statistical differences were observed between control cells and those stimulated with Hp PG+NOD1 (*, P<0.05). (7.83 MB TIF) [file pone.0005396.s003.tif]
